# Reconstruction and Validation of a Genome-Scale Metabolic Model of *Streptococcus oralis* (iCJ415), a Human Commensal and Opportunistic Pathogen

**DOI:** 10.3389/fgene.2020.00116

**Published:** 2020-03-03

**Authors:** Christian S. Jensen, Charles J. Norsigian, Xin Fang, Xiaohui C. Nielsen, Jens Jørgen Christensen, Bernhard O. Palsson, Jonathan M. Monk

**Affiliations:** ^1^ The Regional Department of Clinical Microbiology, Region Zealand, Slagelse, Denmark; ^2^ Department of Bioengineering, University of California, San Diego, San Diego, CA, United States; ^3^ Institute of Clinical Medicine, University of Copenhagen, Copenhagen, Denmark; ^4^ Novo Nordisk Foundation Center for Biosustainability, Technical University of Denmark, Lyngby, Denmark

**Keywords:** *Streptococcus oralis*, mitis group of streptococci, genome-scale reconstruction, constraint-based modeling, Biolog phenotypic profiling

## Abstract

The mitis group of streptococci (MGS) is a member of the healthy human microbiome in the oral cavity and upper respiratory tract. Troublingly, some MGS are able to escape this niche and cause infective endocarditis, a severe and devastating disease. Genome-scale models have been shown to be valuable in investigating metabolism of bacteria. Here we present the first genome-scale model, iCJ415, for *Streptococcus oralis* SK141. We validated the model using gene essentiality and amino acid auxotrophy data from closely related species. iCJ415 has 71-76% accuracy in predicting gene essentiality and 85% accuracy in predicting amino acid auxotrophy. Further, the phenotype of *S. oralis* was tested using the Biolog Phenotype microarrays, giving iCJ415 a 82% accuracy in predicting carbon sources. iCJ415 can be used to explore the metabolic differences within the MGS, and to explore the complicated metabolic interactions between different species in the human oral cavity.

## Introduction

The mitis group of streptococci (MGS) consists of 20 different species, all gram positive cocci arranged as pairs or in chains (Spellerberg and Brandt, 2008). MGS is a part of the lactic acid bacteria, which along with the human pathogenic genera *Streptococcus*, *Enterococcus,* and *Aerococcus* is composed of a wide variety of bacteria important for the food industry (Wu et al., 2017).

Most species in the MGS are considered commensal inhabitants of the oral cavity. These bacteria exist in a complex metabolic relationship with other oral streptococci, as well as other species residing in the oral cavity. These interactions are thought to have an influence on the development of certain oral diseases in humans, e.g., caries (Kreth et al., 2009; Nascimento et al., 2009; Abranches et al., 2018). Sometimes these MGS are able to escape their oral niche and cause an infection of the heart valves, infective endocarditis (IE), a severe disease with a high mortality even with proper treatment. Of all the IE causing species in the MGS, *Streptococcus oralis* is the most common (Rasmussen et al., 2016).

Genome-scale models (GEMs) of metabolism have proven to be valuable in investigating the metabolism in a single bacterial strain (McCloskey et al., 2013). GEMs have been used to compare metabolism between different strains of the same species and between different species (Monk et al., 2013; Ong et al., 2014; Bosi et al., 2016; Monk et al., 2017; Norsigian et al., 2018). When a GEM is used to compare different strains, it is possible to compare the metabolic capabilities with known phenotypic or virulence characteristics, enabling us to identify the metabolic capabilities that either enhance the virulence of a strain or is necessary for a species to be pathogenic (Monk et al., 2013). This provides an unprecedented opportunity to investigate complex mechanisms behind pathogenesis.

Given the implications of *Lactococcus*, *Lactobacillus,* and *Streptococcus* for the food industry, GEMs have been created and published (Oliveira et al., 2005; Teusink et al., 2006; Pastink et al., 2009; Thiele and Palsson, 2010). However, only two GEMs for human pathogenic streptococci have been published so far. One is for *Streptococcus pyogenes*, a distant relative of the MGS with a very different disease spectrum (Jensen and Kilian, 2012; Levering et al., 2016). The other published GEM is for a closer relative, *Streptococcus pneumoniae* (Dias et al., 2019). Although phylogenetically closely related, *S. oralis* and *S. pneumoniae* have several differences. *S. pneumoniae* is a part of the throat microbiome, while *S. oralis is commensal in the* oral microbiome. Furthermore, *S. pneumoniae* is a common cause of pneumonia, otitis media and meningitis. These diseases are only very rarely caused by *S. oralis*.

In this article we present the first GEM for a *S. oralis* strain, named iCJ415. This GEM is based on the genome sequence of *S. oralis* SK141 combined with experimental data using the Biolog phenotypic array or retrieved in published literature. The model is validated with experimental data obtained using either *S. oralis* SK141 or other closely related species in the MGS. This model is intended as a starting point for exploring the complicated metabolic interactions between different bacterial species in the human oral microbiome and for exploring the differences between pathogenic and non-pathogenic bacteria being a part of the human microbiome.

## Materials and Methods

### Strains

GEMs are built using an annotated genome sequence combined with experimental data. We used the strain *S. oralis* SK141, isolated from human dental biofilm (Kilian et al., 2014). The SK141 genome sequence was downloaded from the National Center for Biotechnology Information (https://www.ncbi.nlm.nih.gov/, accession number: JPGA00000000.1). The genome sequence was annotated using the RAST server on default settings (Aziz et al., 2008).

### Reconstruction

The reconstruction was made using the protocol made by Thiele and Palsson (2010). In short, we used the genome annotation to generate a draft reconstruction using Modelseed (Henry et al., 2010). This draft reconstruction was intensively manually curated using the databases BiGG (King et al., 2016), KeGG (Ogata et al., 1999), and MetaNetx (Ganter et al., 2013). If possible, reactions were verified with experimental data. If there was no available *S. oralis* specific data, we used data from closely related species (e.g. *Streptococcus mitis* or *S. pneumoniae*). For gapfilling, pathways were visualized using Escher (King et al., 2015). To identify missing genes, BLASTn homology searches were done using the NCBI webserver with default settings (Boratyn et al., 2013). To ensure a standardized nomenclature and to make comparison between other GEMs easier, all reactions and metabolites in the model were named with BiGG IDs if possible. BiGG Models is a knowledge base now incorporating more than 70 published GEMs (King et al., 2016).

The reconstruction was converted to a mathematical model and all simulations were done, using Constraint-Based Metabolic Modeling in Python (COBRApy) version 0.13.4 (Ebrahim et al., 2013).

### Biolog

Biolog experiments were performed using Biolog’s phenotype microarrays (Biolog, Hayward, CA, USA). All experiments were carried out according to the phenotype microarray protocol for *S. agalactiae* and other *Streptococcus* species (version: 16-May-09) provided by the manufacturer with the following exception: Due to a high degree of background noise in the negative control well, phenotype microarrays 3 was tested using only 1/10 of the carbon sources reported.

The Biolog results were analyzed with the “OPM” package in R, and statistical analysis was done with the “Dunnett-type comparison: one-against-all” function (Vaas et al., 2013). All wells with a P-value < 0.05 when compared with the negative control were considered as having growth.

### Biomass Reaction

The biomass reaction should include all essential constituents, and their fraction, in the biomass composition. Due to the lack of a detailed biomass composition of *S. oralis* we used the macromolecular composition of a *Lactobacillus plantarum* previously published (Teusink et al., 2006). We used the Python package BOFdat (Lachance et al., 2019) to calculate coefficients for DNA, RNA, proteins, lipids, coenzymes and ions. BOFdat divides the biomass calculations into different steps. Step 1 calculates biomass coefficients for DNA, RNA, proteins, and lipids. For determining coefficients for the nucleotides in DNA, it calculates the relative abundance of each nucleotide based on the genome provided. For RNA and amino acid calculations BOFdat uses transcriptomics and proteomics data, respectively. BOFdat uses these data to compare the frequency of RNA and proteins found in these experimental data to calculate the coefficients for nucleotides and amino acids present in RNA and proteins. The data used to RNA and amino acid calculations were obtained from *S. pneumoniae* strain D39 (Aprianto et al., 2018; Sun et al., 2011). To calculate lipids, BOFdat uses the relative abundance, found in experimental data, and the molecular weight of these lipids in the model. We used the lipid composition data from a previously published *Lactococcus lactis* model iAO358 (Oliveira et al., 2005). BOFdat step 2 adds coenzymes and inorganic ions to the model.

Since Gram positive bacteria, in contrast to Gram negative, contains a large amount of peptidoglycan, teichoic acid and lipoteichoic acid, we calculated the coefficients for these structures using data from a *L. plantarum* (Teusink et al., 2006). Further we used data from Xavier et al. to include the following coenzymes considered essential when constructing a metabolic model: Nad, nadp, S-Adenosyl-L-methionine, fad, pyridoxal 5′-phosphate, poenzyme A, tetrahydrofolate, methyltetrahydrofolate, formyltetrahydrofolate, thiamine diphosphate, and riboflavin-5-phosphate (Xavier et al., 2017). The individual contribution of these coenzymes was evenly distributed among the remaining part of the biomass.

All data used for BOFdat and the calculations for metabolites not found by BOFdat are summarized in **Supplementary Table S1**.

## Validation of iCJ415

### Gene Essentiality

No gene essentiality data exists for *S. oralis*, therefore comparison between iCJ415 and experimental gene essentiality data was done using data from the closely related species *S. pneumoniae* (Strain TIGR4) (van Opijnen et al., 2009) and *Streptococcus sanguinis* (Strain SK36) (Xu et al., 2011). Orthologous genes were found using NCBI Bidirectional Blast. COBRApy contains a function, which allows all genes in the model to be deleted individually, and for each gene deletion growth is assessed. This function is similar to *in vivo* experiments of tn-seq and transposon mutagenesis experiments (van Opijnen et al., 2009; Kleckner et al., 1977). Since all gene essentiality experiments were carried out in rich media (e.g. Broth), the simulations were done with all exchange reactions open.

### Amino Acid Auxotrophy

Amino acid auxotrophies were found using data from *S. pneumoniae* strain D39 (Härtel et al., 2012). To compare amino acid auxotrophies found in the experimental data and iCJ415 we opened the exchange reactions corresponding to all metabolites present in the CDM media used to obtain the experimental data. When simulating amino acid omissions we sequentially closed the exchange reactions for the amino acid omitted and tested for growth of the model.

### Carbon and Nitrogen Sources

When testing for carbon sources we found a minimal media in the literature, which did not support *in silico* growth of *S. oralis* without an added carbon source (Homer et al., 1993; Paixão et al., 2015). We used this media and sequentially opened the exchange reaction for the carbon sources and tested for growth of iCJ415. Only Biolog metabolites readily mapped to metabolites in iCJ415 or positive in the Biolog phenotypic system were included.

Nitrogen sources were tested with the same media used to test carbon sources. We used glucose (Opened the EX_glc__D_e exchange reaction) as a carbon source. We sequentially opened and closed the exchange for all nitrogens sources tested. If the opening resulted in an increase in Biomass yield, it was considered a positive result.

All simulations on gene essentiality, amino acid auxotrophy and carbon and nitrogen sources were done using Flux Balance Analysis (FBA) while optimizing for the Biomass reaction. For all metabolites present in the media, the upper and lower bounds were set to -1000 and 1000 mmol/g/h, respectively. When doing Biolog validations the exchange reactions for the carbon- and nitrogen sources tested were also set to -1000 and 1000 mmol/g/h for lower and upper bounds, respectively. **Supplementary Table S2** shows the media content used in the simulations for carbon- and nitrogen sources and amino acid auxotrophies.

For all FBA simulations we used a value of 10^-8^ as a growth/no growth cutoff.

### Biomass Yield

To test the biomass yield and byproduct production, we compared with previously published experimental data obtained in the *S. pneumoniae* strain D39 (Paixão et al., 2015). The D39 strain was grown in a chemically defined media, using four different carbon sources, and the byproducts were measured. These data have been used to validate a recently published *S. pneumoniae* GEM, iDS372 (Dias et al., 2019). We used the same media conditions to simulate growth of iCJ415 (see **Supplementary Table S2**). To simulate the repression of genes by the carbon catabolite repressor A (CcpA) present in MGS, we added additional constraints of the reactions catalyzed by genes repressed by the CcpA, This was done by testing the flux through the reactions catalyzed by the genes repressed by the CcpA using only the constraints imposed by the media. Fluxes through the reactions were then set to 0% (LACOX) or a maximum of 10% of the unconstrained value (ACKr, ALCD2x, PTAr). Due to alleviation of the repression when grown on galactose ACKr, ALCD2x and PTAr were set to a maximum of 100% of the unconstrained value. For the pfl gene, and associated reaction, Dias et al. simulated an underexpression by testing the growth rate and byproduct formation with fluxes from 0-100% of the unconstrained value. For comparison with iDS371, we tested only the value of pfl underexpression giving the best match with experimental data.

These calculations were visualized and calculated using the Escher FBA webapplication (Rowe et al., 2018).

### Memote Test

iCJ415 was tested using memote, which provides a platform to test the performance of a metabolic model (Lieven et al., 2018).

## Results and Discussion

**Figure 1:**
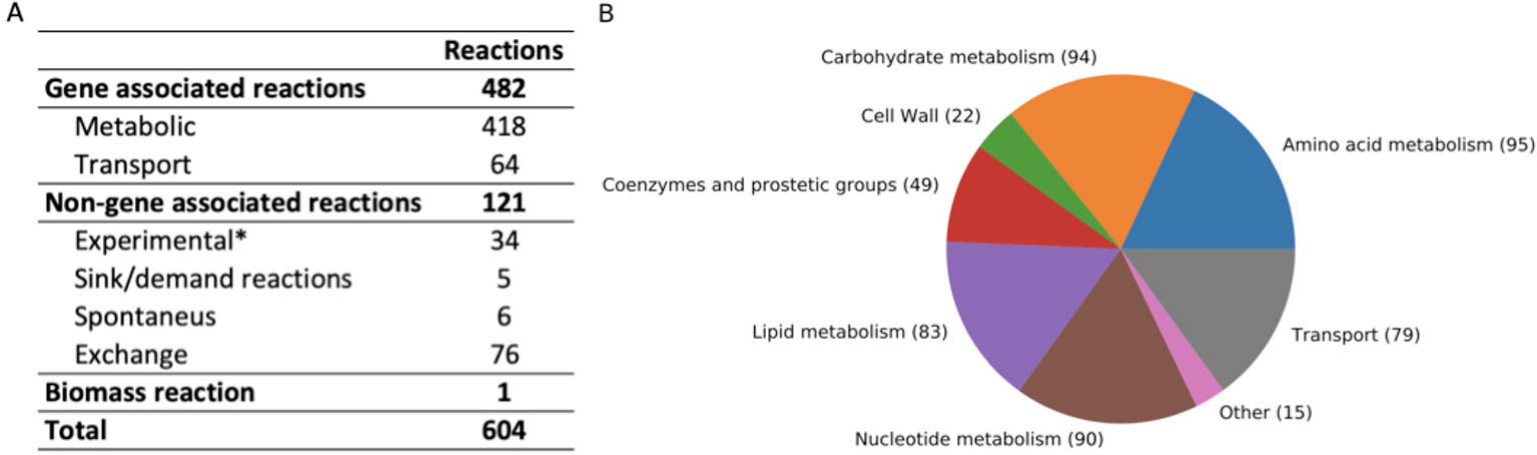
**(A)** Summary of reaction characteristics in iCJ415. **(B)** Reactions in iCJ415 sorted by pathway. Exchange reactions are left out. The reaction pathways are merged together according based on pathways and according to https://www.kegg.jp/kegg/pathway.html. *Based on experimental data, either found in literature or found in Biolog experiments.

The iCJ415 *S. oralis* metabolic model contains 604 reactions, 504 metabolites, and 415 genes. All reactions and metabolites in iCJ415 are detailed in **Supplementary Table S3** and can be found in json and sbml format in **Supplementary Data Sheets 1** and **2.** Of those 604 reactions, 475 are included in the BiGG database, 52 are not included in the BiGG database, and the remaining 76 (excluding the biomass reaction) are exchange reactions. **Figure 1** shows the characteristics of the reactions included in iCJ415. Of the non-gene associated reactions, six are spontaneous and four are added as sink or demand reactions due to lack of available data (e.g., the reaction “Skgcald”) or due to the need for a non-metabolic metabolite in the model, e.g., a sink reaction for DNA. Three reactions were added as gapfilling and 37 reactions were added based solely on biochemical information retrieved either from the literature or Biolog experiments, with no information on the associated gene. We were not able to find the genes responsible for making unsaturated fatty acids, and we added these based only on data present in literature. The same was the case for the reaction “UDPACGALRIBGALUDPAATGALS,” which produces one of the last reactions in the teichoic acid synthesis. The gene responsible for this reaction was not found, but we added the reaction based on experimental data (Denapaite et al., 2012). These non-gene associated reactions are placed in a separate sheet in **Supplementary Table S3**.

### Biomass Reaction

Using the software BOFdat we constructed a biomass reaction using genomic, transcriptomic, proteomic and lipidomic data from either *S. oralis* SK141 or closely related species. When comparing with another GEM from a closely related species, *S. pneumoniae*, iDS372, the biomass reactions have many similarities, though there are differences. In contrast to iDS372 we decided not to include polyamines into the Biomass reaction of iCJ415, despite polyamines has been shown to be import in nasopharyngeal carriage, and virulence of *S. pneumoniae*. This is because *S. oralis* does not colonize the nasopharynx and is usually not considered a pneumonia-causing agent like *S. pneumoniae* (Shah et al., 2011). The biomass reaction of iCJ415 is also similar to that of a S. pyogenes GEM (Levering et al., 2016). However, *S. pyogenes*, contains a capsule while *S. oralis* SK141 does not. Therefore we did not include a capsule in the biomass reaction of S. oralis SK141 (Levering et al., 2016).

### Verification of iCJ415 Using Experimental Essentiality Data

**Figure 2:**
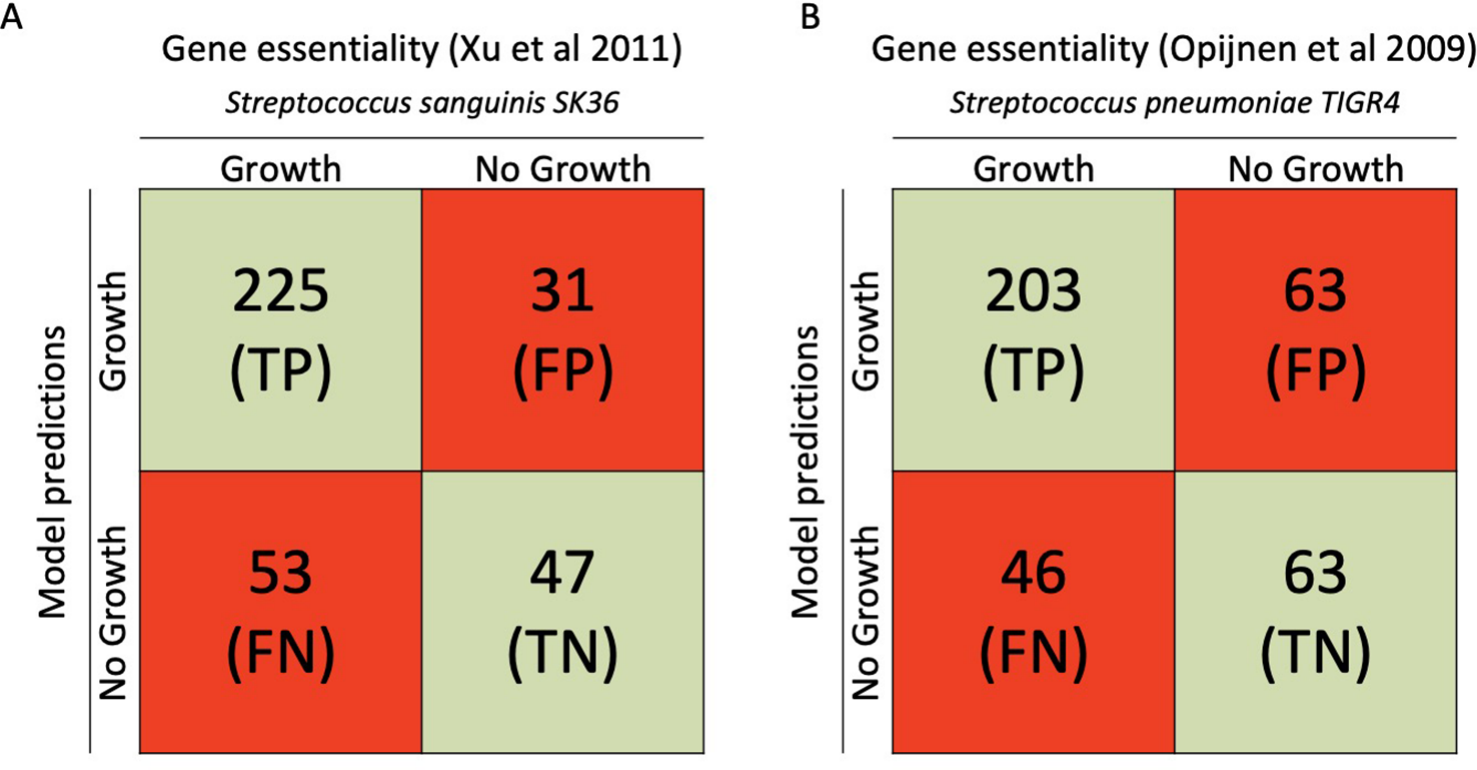
Gene essentiality comparison between iCJ415 and experimental data. **(A)** When iCJ415 is compared with *S. sanguinis* SK36 there is a 76% (272/356) concordance in gene essentiality. **(B)** When comparing iCJ415 with *S. pneumoniae* TIGR4 tn seq data there is a 71% (266/375) concordance. Gene orthologs in *S. sanguinis* SK36 and *S. pneumoniae* TIGR4 were found using bidirectional blast.

Gene essentiality analysis is an important measure for validating the model, especially when considering new possible antimicrobial drug targets. We predicted a list of genes that are essential for growth using COBRApy to do a single gene knockout simulation in iCJ415. By using data from the closely related bacteria *S. sanguinis* and *S. pneumoniae*, Bidirectional Blast Hits found 356 and 375 orthologous genes in *S. oralis* SK141, respectively. The gene essentiality predictions in iCJ415 matched the *S. sanguinis* and *S. pneumoniae* gene essentiality datasets in 76% and 71% of the cases, respectively (See **Figure 2**, **Supplementary Table S4**).

When comparing the gene essentiality datasets from *S. sanguinis* and *S. pneumoniae*, 336 of the genes in iCJ415 were found in both essentiality datasets and 79 were found in only one, or in none of the datasets. Discrepancy between the two essentiality datasets were observed with 62 genes. These discrepancies can partly be explained by how the experimental data were obtained. The *S. pneumoniae* essentiality data were from a tn-seq experiment and the *S. sanguinis* data were from gene knockout mutants.

Since we are using two gene essentiality dataset from two closely related bacteria, we only look at those genes where the experimental gene essentiality data agrees. Genes are termed “concordant,” when both gene essentiality datasets and iCJ415 results are the same and “discordant” when the gene essentiality datasets are the same, but they are different from gene essentiality predictions in iCJ415. The terms discordant and concordant are also used in **Supplementary Table S4** to describe the two situations. **Table 1** shows all the concordant and discordant genes categorized on pathway. Most pathways are almost equally represented in the concordant and discordant group, though amino acid metabolism has a higher representation in the discordant group and transport has a higher representation in the concordant group. It is worth noticing that both peptidoglycan and terpenoid backbone synthesis are highly represented in the concordant group. They are both simple synthetic pathways with only one gene responsible for each reaction, and the end product is essential for growth of the bacteria. This is in opposition to the amino acid metabolism where several reactions can lead to the same product.

**Table 1:** Concordant and discordant genes in iCJ415 categorized according to subsystem.

	Concordant (c)		Discordant	
Subsystem (b)	genes, no	genes, % (a)	genes, no	genes, % (a)
Amino acid metabolism	37	17	23	37
Carbohydrate metabolism	37	17	12	20
Lipid metabolism	13	6	4	7
Metabolism of cofactors and vitamins	13	6	6	10
Nucleotide metabolism	40	19	11	18
Peptidoglycan biosynthesis	8	4	0	0
Terpenoid backbone synthesis	7	3	1	2
Transport	50	23	4	7
Other	7	3	0	0
Total	212		61	

(a) Calculated as the proportion concordant/discordant genes as a total of concordant/discordant genes.

(b) Subsystem designation are derived from **Supplementary Table S3** but are merged together into groups according to the BiGG website (https://www.kegg.jp/kegg/pathway.html).

(c) Discordant refers to genes where experimental data show agreement but is different from iCJ415 predictions. Concordant refers to genes where experimental data shows agreement and is similar to iCJ415 predictions.

There are 61 genes where there are discordant results. In 36 of those genes, iCJ415 shows essentiality while the essentiality datasets show non-essentiality. An explanation is that not much *S. oralis* specific data is available, which causes a lack of alternative genes or pathways in the model. This leads to genes being essential *in silico*, while they are not essential *in vitro*. This is the case with all but one of the amino acid genes (SK141_RS08355) categorized as discordant.

In the remaining 25 genes where there are discordant results, iCJ415 shows genes to be non-essential, while the essentiality data shows the genes to be essential. This can be explained in part by the use of rich medium in both gene essentiality datasets. Both of these studies use a broth-based, non-defined media, where it is not possible to get the exact composition of the different metabolites. When we simulate gene deletions in rich media we open all exchange reactions in the model, which corresponds to an experimental setup where all metabolites are present in the growth media. But if a metabolite is absent from the experimental growth media, a gene could be deemed essential, however the gene would not be essential if the metabolite was present.

The two gene essentiality studies use either Todd Hewitt or BHI media for the experiments (van Opijnen et al., 2009; Xu et al., 2011). Both of these media contain ingredients such as digests of beef-heart, neopeptones, brain, or gelatin. These ingredients make the exact composition of the media unknown, and they will probably differ between batches and manufacturer. Despite the unspecific media conditions, both Todd Hewitt and BHI have added glucose as a carbon source, which probably make it the primary carbon source available (Atlas and Snyder, 2019). Therefore we tried to do the gene essentiality calculations for all discordant genes from **Supplementary Table S4**, leaving out all carbon sources except glucose. See **Table 2** for discordant genes that are non-essential in iCJ415, but essential in the experimental essentiality data. Furthermore, **Table 2** shows differences in essentiality and growth rate when grown in full media, compared with glucose as the sole carbon source. The growth calculations are percent of growth when gene is not knocked out.

**Table 2:** Discordant genes that shows non-essentiality in iCJ415 *in-silico*, while they were shown to be essential in both experimental datasets.

System	Gene	Reactions	Growth, full media (a)	Growth, glucose (b)	Comment
Amino acid metabolism	SK141_RS08355	GF6PTA	100,00	0,00	There are several reactions which can produce gam6p, including N-Acetyl-D-glucosamine/D-Glucosamine PTS transporters, if it is present in the media.
Carbohydrate metabolism	SK141_RS00135	PRPPS	100,00	100,00	5-Phospho-alpha-D-ribose 1-diphosphate can also be produced in the reaction UPPRT.
	SK141_RS00605	GALUi	99,74	0,00	When galactose is present in the media, UDP-glucose can be produced without SK141_RS00605, making SK141_RS00605 non-essential.
	SK141_RS00635	PGI	84,82	74,59	Fructose 6-phosphate can be produced using the pentose phosphate pathway, though less efficient than through PGI.
	SK141_RS00905	GAPD	58,69	55,00	As with PGK, there are an alternative route for 3-Phospho-D-glycerate production.
	SK141_RS01515	PGK	58,69	55,00	there is an alternative route from Glyceraldehyde 3-phosphate to 3-Phospho-D-glycerate *via* the reactions G3POR
	SK141_RS01855	TPI	58,68	0,00	When grown in glucose only, there is a lack of Phosphoenolpyruvate to use in the transporter when the gene is knocked out.
	SK141_RS01965	FBA2, FBA3, FBA	84,95	0,00	As with TPI there is lack of Phosphoenolpyruvate
	SK141_RS03865	G6PDH2r	100,00	0,00	When only grown on glucose, teichoic acid cannot be produced due to a lack of D-Ribulose 5-phosphate.
	SK141_RS05000	PYK	85,69	58,55	There are other, not as effective, sources of pyruvate in the model.
	SK141_RS05005	PFK_2, PFK, PFK_3	84,95	0,00	When grown on multiple carbon sources D-Fructose 1,6-bisphosphate can be produced using several carbohydrates.
	SK141_RS07980	GND	100,00	0,00	When growing on glucose the only way to produce teichoic acid is through the GND reaction.
Metabolism of cofactors and vitamins	SK141_RS06615	ACPS1	100,00	100,00	In a steady state this gene is not needed, since ACP is not included in the biomass reaction.
	SK141_RS06335	FOLR2, DHFR	100,00	100,00	S. Oralis SK 141 has two genes annotated for these reactions, making them non-essential
	SK141_RS06020	NADS1	100,00	100,00	S. Oralis SK141 has an alternative Nicotinamide adenine dinucleotide producing reaction, NMNAT.
	SK141_RS01060	THFGLUS, DHFS	100,00	100,00	S. Oralis SK 141 has two genes annotated for these reactions, making them one non-essential
Nucleotide metabolism	SK141_RS04680	RNDR2, RNDR3, RNDR4, RNDR1	100,00	100,00	S. Oralis SK141 contains another gene annotated capable of producing deoxynucleotides (SK141_RS08300)
	SK141_RS04685		100,00	100,00	
	SK141_RS04880	ATPS4r	99,80	99,91	This gene is not used for ATP synthesis in Streptococcus, but is probablys primarily used for keeping the internal h+ homeostasis(c).
	SK141_RS04885		99,80	99,91	
	SK141_RS04890		99,80	99,91	
	SK141_RS04895		99,80	99,91	
	SK141_RS04900		99,80	99,91	
	SK141_RS04905		99,80	99,91	
	SK141_RS04910		99,80	99,91	

(a) Growth when all exchange reactions are set to the upper and lower bound default values of 1,000 and -1,000. Growth are calculated as percent of value when gene is knocked out, compared with no gene knock-out.

(b) Growth when all carbon source exchange reactions, except glucose, are closed. All other exchange reactions, and glucose, are set to upper and lower values of -1,000 and 1,000, respectively.

(c) See PMID 30930283.

When using glucose as a sole carbon source, seven “discordant” genes become essential; six of those are part of the carbon metabolism. These genes are becoming essential due to the lack of essential metabolite synthesis when only glucose is used as a carbon source, e.g. teichoic acid cannot be produced when the gene SK141_RS03865 is knocked out due to the inability of the model to synthesize ribulose 5-phosphate. When retesting all genes that were “concordant” in the full media, only one of the 169 genes were essential when testing with glucose as the sole carbon source. Furthermore, only 10 gene deletions had a lower growth rate when glucose was the only carbon source, and no deletion gave more than a 20% reduction in growth rate.

When publishing iDS372, the authors did gene essentiality testing in iDS372 and compared with essentiality data in the Online Gene Essentiality Database using a chemically defined media, despite the experimental essentiality data was generated in a complex media (Chen et al., 2012; Dias et al., 2019). We tested the gene essentiality of iDS372 by opening all exchanges (Simulating rich media conditions) and did gene knockout using COBRApy. Thirteen genes, which showed concordance between iCJ415 and the experimental data, did not show concordance in iDS372. Thirty-three genes that showed discordance between iCJ415 and experimental data showed concordance in iDS372. These differences can be explained, by that none of the essentiality datasets is from a *S. oralis*. Further, *S. oralis* has one of the smallest genomes in the MGS, having a genome size of 1.9 mio basepairs (Rasmussen et al., 2016). In contrast, both *S. pneumoniae* and *S. sanguinis* have larger genome sizes of around 2.1 and 2.4 mio basepairs, respectively, giving a relative larger amount of genes (Tettelin et al., 2001; Rasmussen et al., 2016). This can lead to alternative pathways, or multiple genes catalyzing the same reaction. The latter is the case in at least nine of the genes showing discordance in iCJ415 but concordance in iDS372.

These results indicate that most gene deletions in iCJ415, give the expected result, but there are still discrepancy between iCJ415 and the experimental data. However, much of the difference can be explained by how the essentiality data were obtained and the use of different species.

The essential genes found in iCJ415, can be used to find potential drug targets. Especially when iCJ415 can be applied to a larger set of strains, essential genes found in all strains could be possible drug targets. To further validate iCJ415, species-specific data would be helpful, preferably obtained in a chemically defined medium.

### Experimental Verification of Carbon- and Nitrogen Utilization in iCJ415

**Figure 3:**
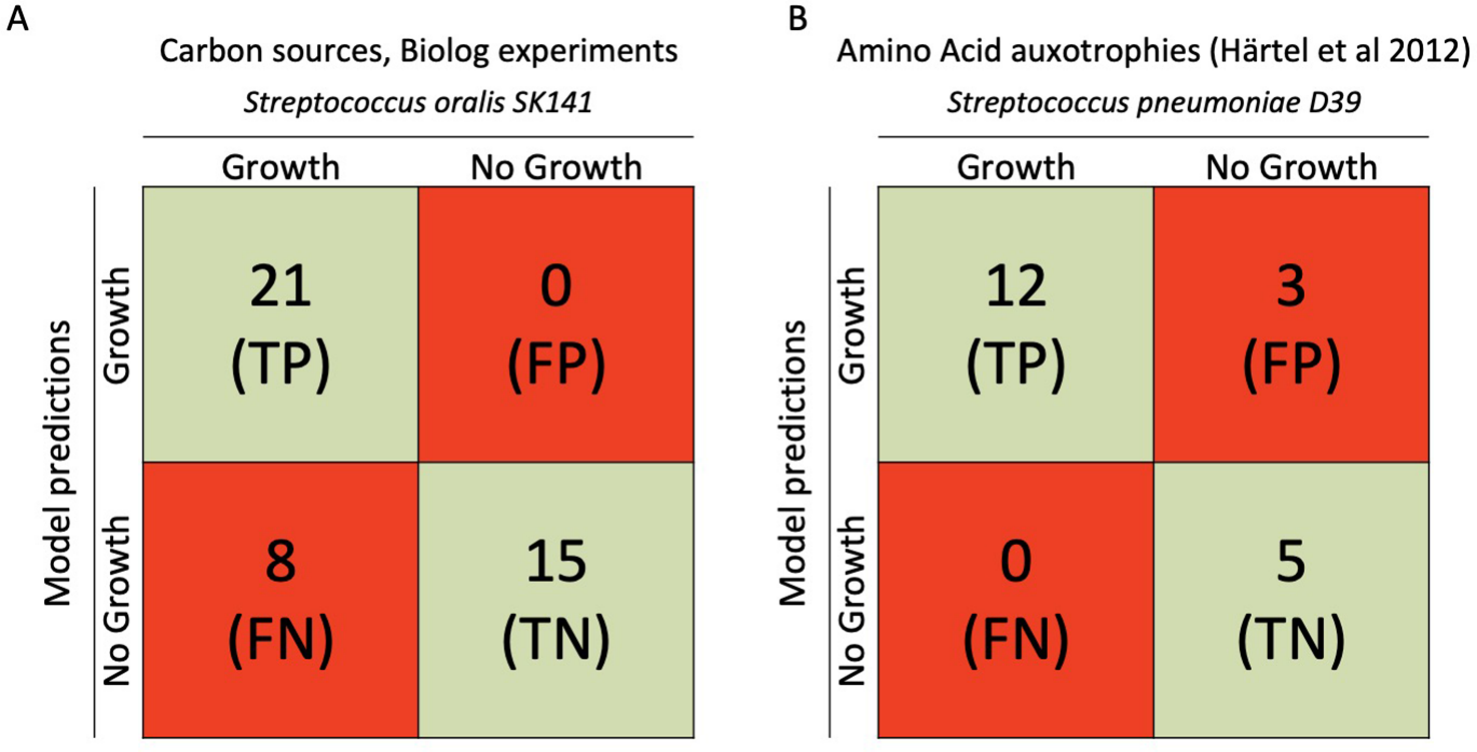
Comparison between iCJ415 and experimental data. **(A)** Comparison between carbon utilization in iCJ415 and data obtained using *S. oralis* SK141 and the Biolog phenotypic array. There is an 84% (36/43) concordance between iCJ415 and Biolog phenotypic array using different carbon sources. **(B)** Amino acid auxotrophy comparison between iCJ415 and experimental data obtained using *S. pneumoniae* D39. There is an 85% (17/20) concordance between iCJ415 and the experimental data.

To investigate *S. oralis* SK141 growth on different Carbon- and nitrogen sources we used the Biolog microarray system. For model simulations we used a minimal media found in the literature (Homer et al., 1993; Paixão et al., 2015). All metabolites mapped to iCJ415 or found to be positive in the Biolog system were tested for growth in iCJ415 (see **Figure 3**, **Supplementary Table S4**). The Biolog results for S. oralis SK141 can be found in **Supplementary Data Sheet S3**.

The Biolog microarray system tests 190 different carbon sources. Of those 190 carbon sources, 44 where either mapped to iCJ415 or were positive in the Biolog microarray system. Though, dextrin and pectin were positive in the Biolog microarray system, they were not included due to the heterogeneity of the two carbon sources.

Knowledge about the differences in carbon source utilization within the MGS is limited. The published data is mainly about *S. pneumoniae* (Leonard and Lalk, 2018; Marion et al., 2012). Therefore it is interesting to compare the differences between *S. oralis* and *S. pneumoniae*. Though, phylogenetically closely related, *S. oralis* and *S. pneumoniae* are part of two different microbiomes in the human body, the oral cavity and upper respiratory airways respectively. Furthermore, their spectrum of disease is very different. *S. pneumoniae* being the etiologic agent of pneumonia, meningitis and otitis media, while *S. oralis* being etiologic agent of endocarditis. Of the 43 carbon sources mapped to iCJ415, eight (18%) were not able to promote growth in iCJ415. Four of them had transport reactions in iCJ415 but were still not able to use the metabolites as carbon sources (glycerol, dihydroxyacetone, deoxyribose, and tagatose). The remaining four metabolites were not present in the model and we could not find any information on the transport or metabolism in the genome annotation or in the literature (propionic acid, 5-keto-D-gluconic acid, oxalomalic acid, sorbic acid).

These metabolic associated genes are increasingly considered crucial to the pathogenesis since they determine in which ecological niche an organism can grow, both during asymptomatic carriage and in disease (Shelburne et al., 2008). Therefore, the variety of the carbon sources a strain is capable of utilizing is thought to be important for the pathogenesis, and can be valuable for understanding which pathogenetic traits are important for specific bacteria under certain conditions. When *S. oralis* and *S. pneumoniae* resides in two different ecological niches, different nutritional sources are available. While the oral cavity contains multiple different carbon sources, partly degraded by oral enzymes, pharynx has a low content of readily available carbon sources (Shelburne et al., 2008; Philips et al., 2003). When comparing genome annotations for *S. pneumoniae* R6 and *S. oralis* SK141 this is reflected by the content of transport systems specific for breakdown products of human tissue. *S. pneumoniae* R6 contains a transporter for hyaluronate, which is not present in *S. oralis* SK141, and together with lyases this transporter can make part of the human extracellular matrix available as carbon sources (Marion et al., 2012). *S. pneumoniae* mutants deficient of these lyases are attenuated in their ability to colonization and infection making them important virulence factors (Manco et al., 2006; King, 2010).

The *S. pneumoniae* and *S. pyogenes* GEMs previously published all contains several carbohydrate transporters, but the hyaluronate transporter was not included in any of them. The GEM for *S. pneumoniae* R6, iDS372, contains transporters for nine carbon sources also present in iCJ415. While iDS371 can grow on six of these carbon sources, iCJ415 can grow on 8. Neither of the models can use glycerol as a carbon despite having a transporter. In contrast to iCJ415, iDS372 cannot grow on either stachyose or raffinose, which is due to lack of a raffinose degrading reaction (Stachyose is degraded to raffinose). Raffinose utilization by *S. pneumoniae* has been shown to be an independent predictor for disease spectrum (Minhas et al., 2019).

Though, Biolog data showed that *S. oralis* SK141 was able to use some nitrogen sources which could be mapped to iCJ415 (D-glucosamine, N-acetyl-D-glucosamine, and N-acetyl-D-mannosamine), we were not able to distinguish single nitrogen capabilities in iCJ415 with the used *in silico* media, revealing that there are still knowledge gaps in iCJ415.

iCJ415 reflects that *S. oralis* is able to grow on multiple carbon sources, but the transport and metabolism of some of these metabolites are unknown or only poorly understood.

### Verification of Amino Acid Auxotrophies in iCJ415 Using Experimental *S. pneumoniae* Data

To evaluate iCJ415 we compared amino acid auxotrophies in iCJ415 with experimental data. By omitting each one of the 20 amino acids in a synthetic chemically defined medium, Härtel et al., found the amino acid auxotrophies for *S. pneumoniae* D39. We opened the exchange reactions for all the amino acids in iCJ415 and all the metabolites present in the media used by Härtel et al. To test amino acid auxotrophy we consecutively closed each of the amino acid exchange reactions in iCJ415 (**Figure 3**, **Supplementary Table S4**). Three amino acids had discrepant results between experimental data and iCJ415.

Isoleucine, valine, and leucine are not auxotrophic in iCJ415 but show auxotrophy in the experimental omission data. In iCJ415 all three amino acids can be synthesized from pyruvate and threonine. All the reactions in these pathways are added based solely on the genome annotations. According to Härtel et al., the genes for the synthesis of the three amino acids are also present in *S. pneumoniae* D39, but are apparently not active under the conditions tested. Whether the same applies for *S. oralis* SK141 is not known.

Like the ability to grow on different carbon sources, amino acid auxotrophies can be an important pathogenetic trait. The ability to grow in the absence of a specific amino acid has been shown to be important for which metabolic niche a bacterium can cause disease. This can be important when a commensal bacteria, like *S. oralis*, causes disease. If the bacterium is auxotrophic for an amino acid not present in the environment, it cannot grow, and hence not cause disease. To exemplify this, it has been shown that the human commensal *Haemophilus influenza*, is more likely to have a histidin synthesizing machinery when isolated from the middle ear in otitis media patients, than when isolated from the throat in healthy humans (Juliao et al., 2007).

### Validating Growth Rate of iCJ415 Using *S. pneumoniae* Data

For validating Biomass yield and byproduct formation of iCJ415 we used previously published experimental growth data from *S. pneumoniae* D39 (Paixão et al., 2015). The catabolite control protein A (CcpA) is used in many bacteria, including streptococci, to organize the carbohydrate catabolism for obtaining maximal growth. These data have previously been used to validate a *Streptococcus pneumoniae* R6 GEM, iDS372 (Dias et al., 2019). To simulate the impact of the carbon catabolite repressor on the genes in the model, fluxes in reactions catalyzed by repressed genes were set to a maximum flux defined by the flux in the unconstrained media, see materials and methods.

**Table 3** shows the results from iCJ415, the results from iDS372 and experimentally obtained data from *S. pneumoniae* D39. The experimental data was obtained when grown in glucose, galactose, N-acetyl-D-glucosamine, and mannose. When compared with iDS372, iCJ415 has a lower growth rate than iDS372 except when N-acetyl-D-glucosamine is used as a carbon source. Especially when using glucose as a carbon source, the growth rate is only 0.44 h^-1^ in iCJ415, with growth rates in iDS372 and in the experimental of 0.85 and 0.82 h^-1^ respectively. When grown on glucose the 0% activity of pfl causes severe growth retardation in iCJ415, but only a small increase to 1% pfl activity causes an increase in growth rate to 0.76 h-1. The reason for decrease in growth rate is due to lack of formate production when the pfl gene is knocked out. Formate is used for the synthesis of folates in the reaction “FTHLFI.” This reveals differences in the synthesis of folates, which in part can be explained by a folate transporter in iDS372. A folate transporter has earlier been identified in *Streptococcus mutans*, but we could not find any evidence for the folate transporter being present in the annotation of *S. oralis* or *S. pneumoniae* and it has been postulated earlier that such a transporter is not present in *S. pneumoniae* (Eudes et al., 2008; Burghout et al., 2013). Due to the lack of evidence, we choose not to include a formate transporter in the model.

**Table 3:** Growth rate and byproduct formation in iCJ415 and an S. Pneumoniae GEM (iDS372). The simulations were done with additional constraints trying to simulate the action of the Carbon Catabolite repressor.

	pfl underexpression	Carbohydrate uptake	Growth rate	Lactate	Formate	Acetate	Ethanol
	%	mmol g−1 h−1	h-1	mmol g−1 h−1	mmol g−1 h−1	mmol g−1 h−1	mmol g−1 h−1
**Glucose**							
iCJ415	0	34.09	0.45	64.9	0	0	0
iDS372	0	34.09	0.85	66.7	0	0	0
Experimental data			0.82	57.52	0	0.9	0.11
**Galactose**							
iCJ415	90	22.6	0.61	4.61	39.6	20.9	16.6
iDS372	90	22.6	0.82	4.1	41.81	20.61	19.15
Experimental data			0.47	3.78	28.67	14.53	13.93
**N-acetyl-D-glucosamine**							
iCJ415	0	35.86	0.92	66.2	0	34.3	0
iDS372	0	35.86	0.89	70.0	0	35.86	0
Experimental data			0.53	61.37	2.03	1.15	0.6
**Mannose**							
iCJ415	10	32.52	0.75	57,2	5.28	3.12	0
iDS372	10	32.52	0.85	58,77	6.8	3.04	1.63
Experimental data			0.41	49,02	5.71	3.0	2.98

*iDS372 results and “Experimental data” adapted from PMID 31293525.

Interestingly, iCJ415, like iDS372, has a much higher rate of acetate production, than the experimental data, when N-acetyl-D-glucosamine is used as a carbon source. The high acetate production I iCJ415 is explained by a production of acetate in the breakdown of N-acetyl-D-glucosamine 6-phosphate. This anomaly reveals a knowledge gap in the metabolism of N-acetyl-glucosamine in *S. oralis*.

### Memote Testing

iCJ415 was tested using the Memote web application with all exchanges open. iCJ415 scores >99% on all consistency scores except “unbounded flux in default medium” where it scores 95% (Lieven et al., 2018).

## Conclusions

The aim of this study was to construct a GEM of a *S. oralis*, the first GEM of a bacterium that is a major constituent of the human oral microbiome and an IE causing bacterium. We used an annotated genome together with strain, species or genus specific data obtained from the literature. To get information in carbon source utilization in *S. oralis* SK141 we used the Biolog phenotypic microarray.

When comparing gene essentiality between iCJ415 and published experimental data, we found concordance in 71–76% of the genes tested. When testing carbon sources and amino acid auxotrophies we found concordance with experimental data in 82% and 85% of metabolites tested, respectively. Especially the carbon source utilization data obtained in this study are valuable. It is, beside the genomic data, the only strain specific data used. Furthermore, carbon source utilization capacities are increasingly being considered as possible virulence factors in bacteria (Leonard and Lalk, 2018; McAllister et al., 2012; Juliao et al., 2007). We found that *S. oralis* are able to grow on at least 28 carbon sources, including commonly known carbohydrates as glucose and galactose, but also rarely encountered ones like lyxose. iCJ415 were able to grow on 21 of the 28 carbon sources tested. In the remaining seven carbon sources we did not have enough information to simulate growth on these carbon sources.

iCJ415 should be used as a starting point for exploring the complicated metabolic interactions between different strains of the same species and between different species in the human mouth. Further it can be used to compare metabolic capabilities between non-pathogenic and known pathogenic strains, finding metabolic traits important for virulence.

iCJ415 was tested using memote and scored >99% on all consistency scores except “unbounded flux in default medium” where it scores 95%.

## Data Availability Statement

All datasets generated for this study are included in the article/**Supplementary Material**.

## Author Contributions

BP, XN, JC, and CJ conceived the project. XF, CN, JM, and CJ performed the metabolic network reconstruction. CN and CJ performed the model simulations. CJ prepared the draft manuscript. All authors have read, commented and approved the final manuscript.

## Funding

This work was supported by the Region Zealand Foundation for Health Research, The Knud Højgård Foundation (Grant no: 18-02-0767), and the Ovesen Foundation. None of the funders had any influence on the study or the conclusions.

## Conflict of Interest

The authors declare that the research was conducted in the absence of any commercial or financial relationships that could be construed as a potential conflict of interest.

## Supplementary Material

Table S1 Biomass calculations.

Table S2 in-silico media contents.

Table S3 Reactions and metabolites in iCJ415.

Table S4 Gene essentiality, amino acid auxotrophies and carbon source testing of iCJ415.

Data Sheet S1 iCJ415 in JSON format.

Data Sheet S2 iCJ415 in SBML format

Data Sheet S3 Biolog results for S. oralis SK141.
