# Supplementary material for: Reconstruction and Validation of a Genome-Scale Metabolic Model of Streptococcus oralis (iCJ415), a Human Commensal and Opportunistic Pathogen
Source: Front Genet. 2020 Mar 3;11:116. doi: 10.3389/fgene.2020.00116 (PMC7063969; doi:10.3389/fgene.2020.00116)

# PM01 (Carbon Sources)

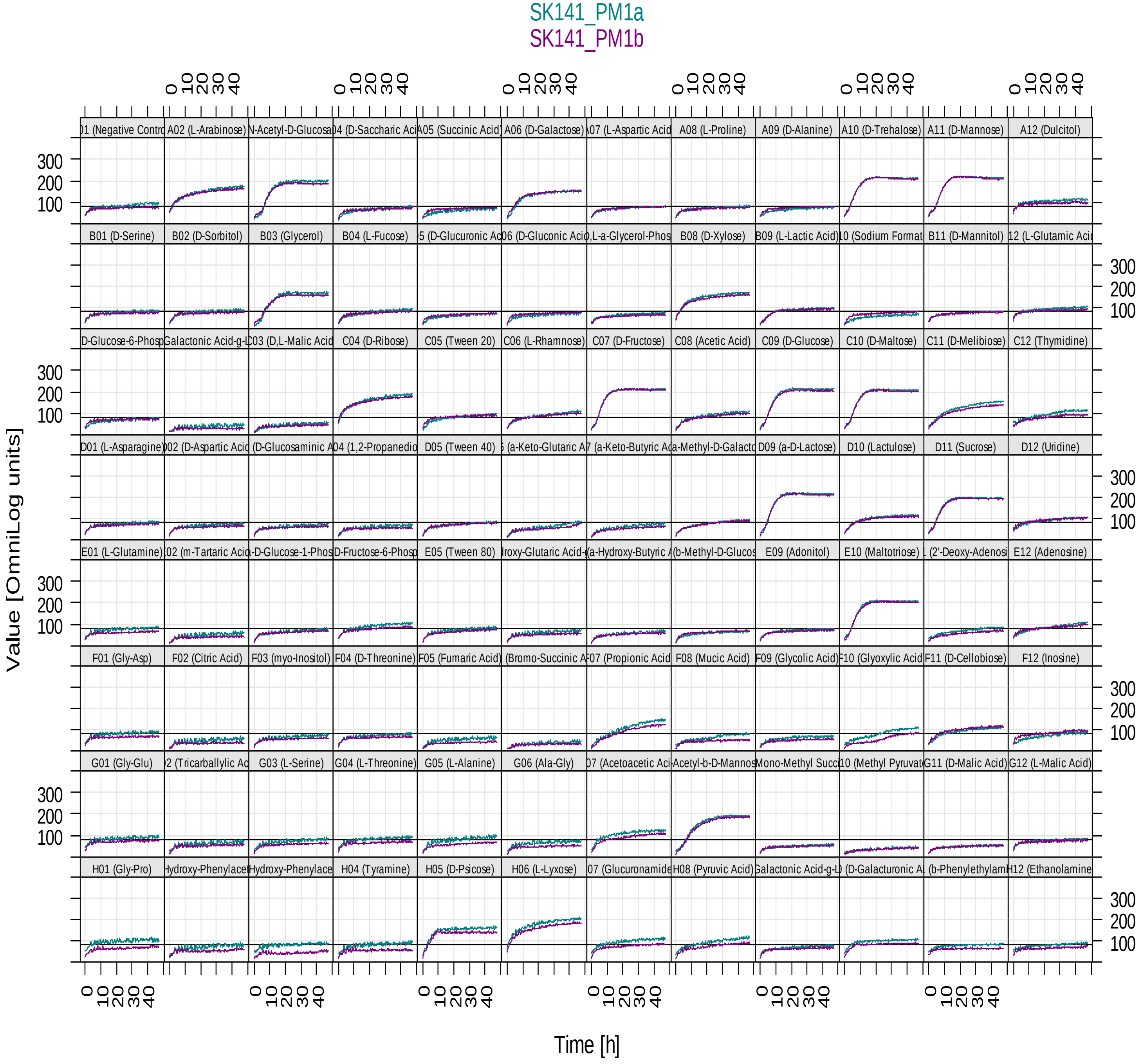

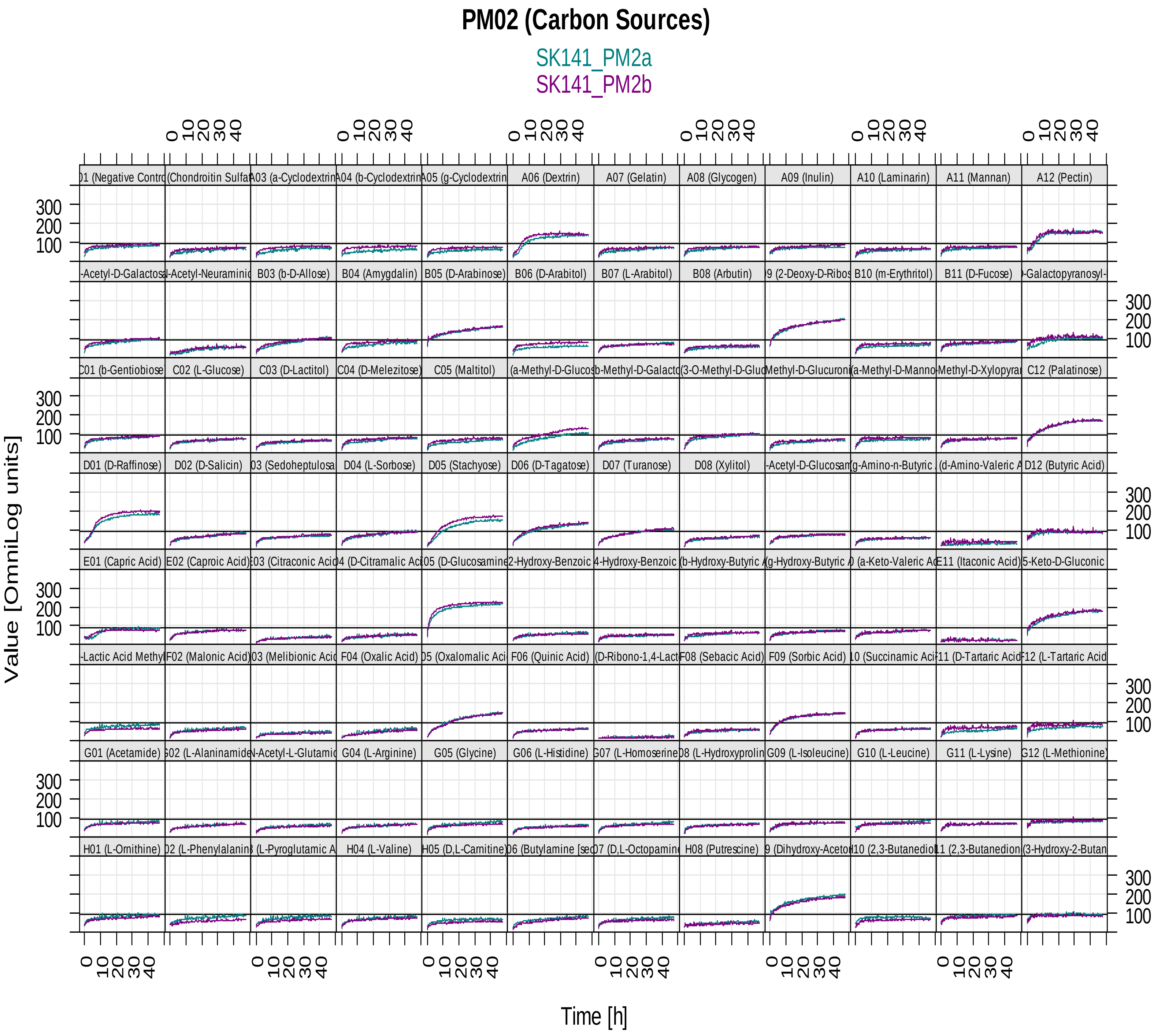
PM02 (Carbon Sources)
SK141_PM2a
SK141_PM2b
Value [OmniLog units]
Time [h]

# PM03 (Nitrogen Sources)

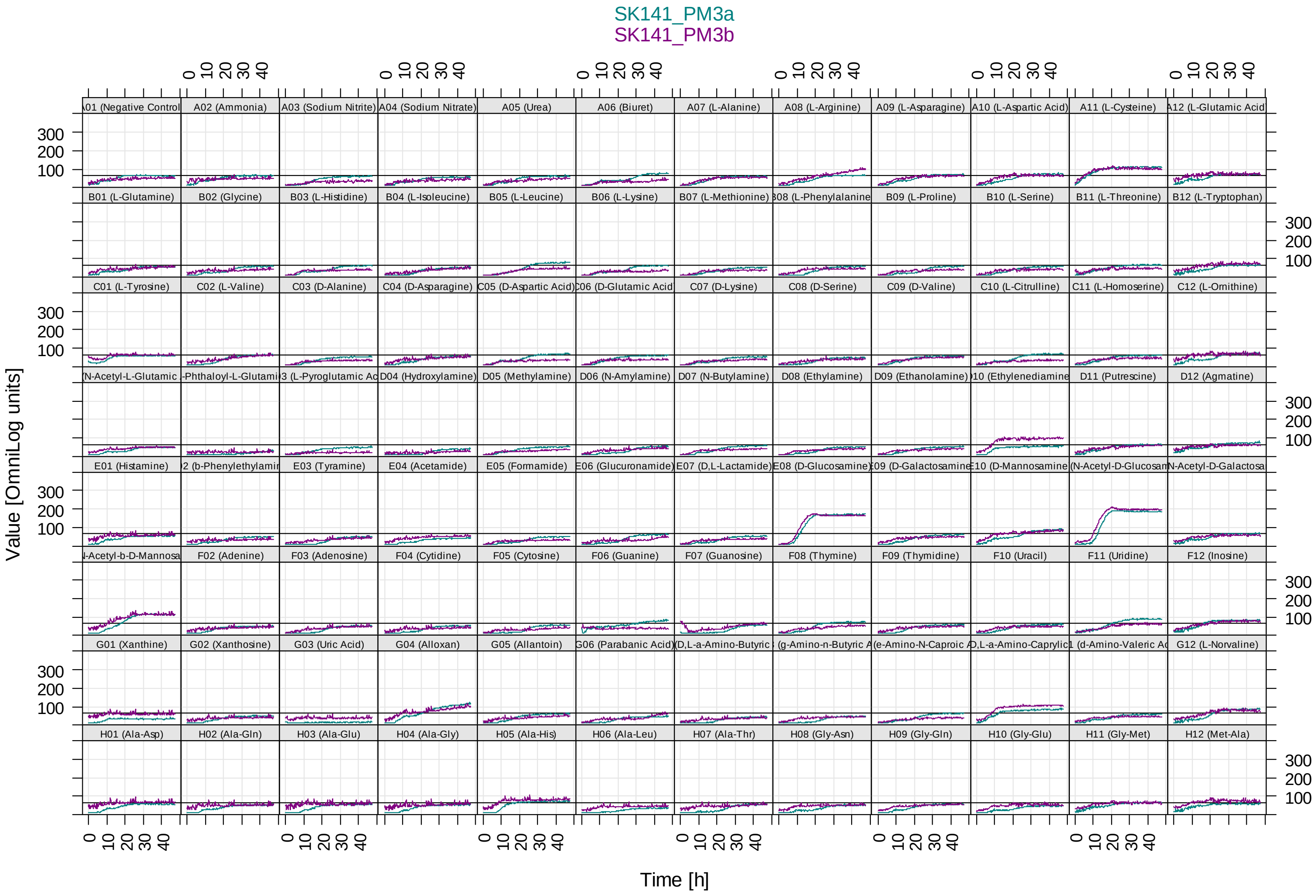

## PM04 (Phosphorus and Sulfur Sources)

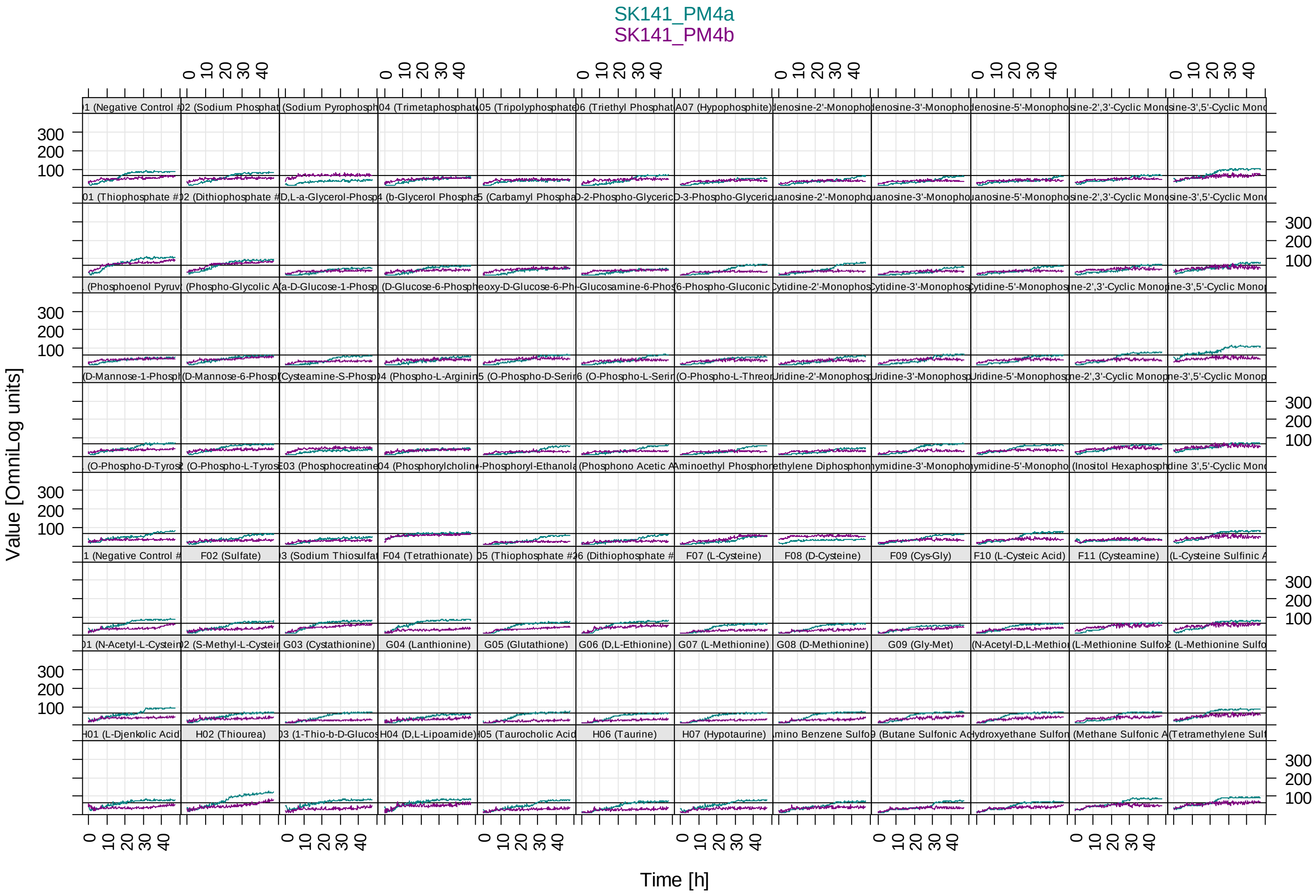

# PM05 (Nutrient Supplements)

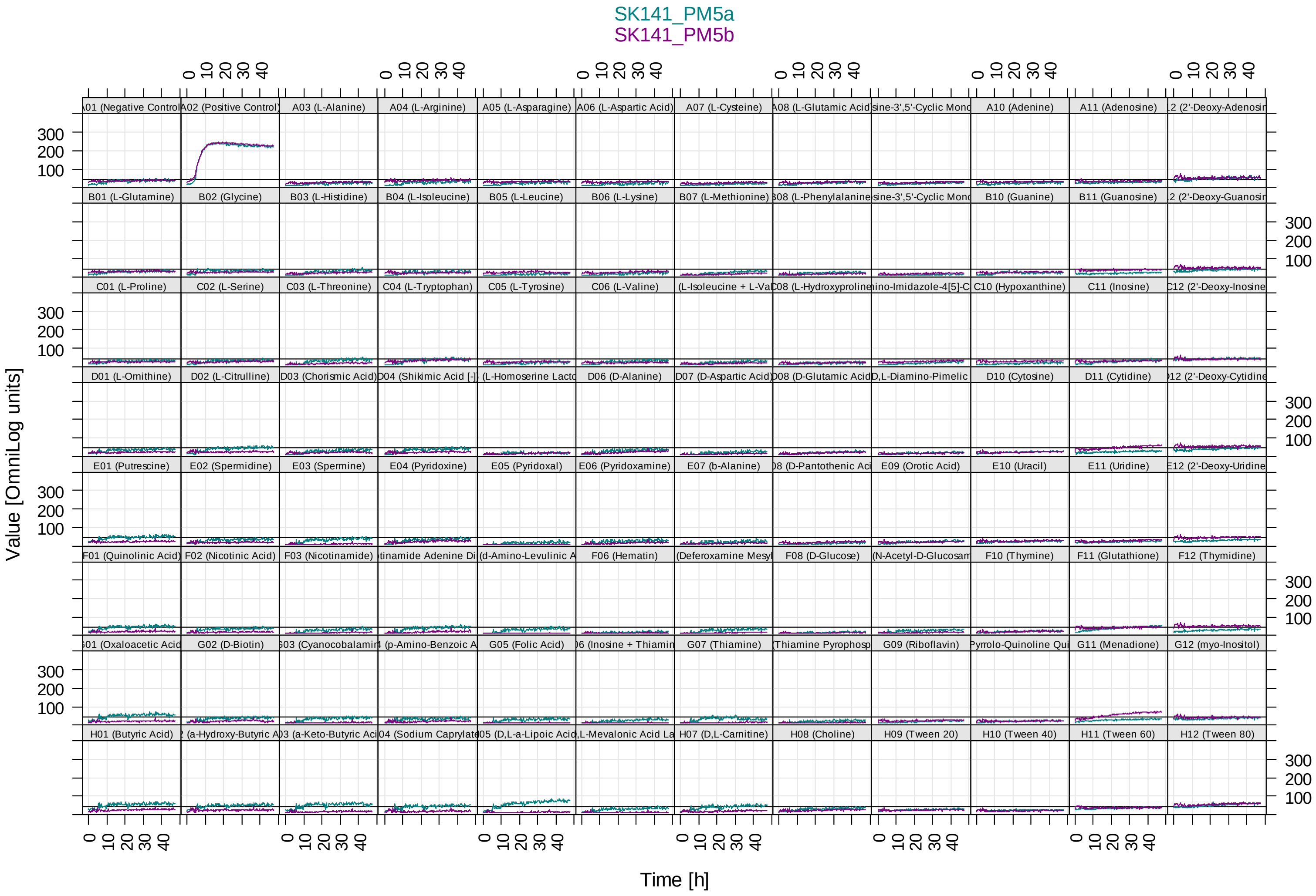

## PM06 (Peptide Nitrogen Sources)

SK141_PM6a
SK141_PM6b

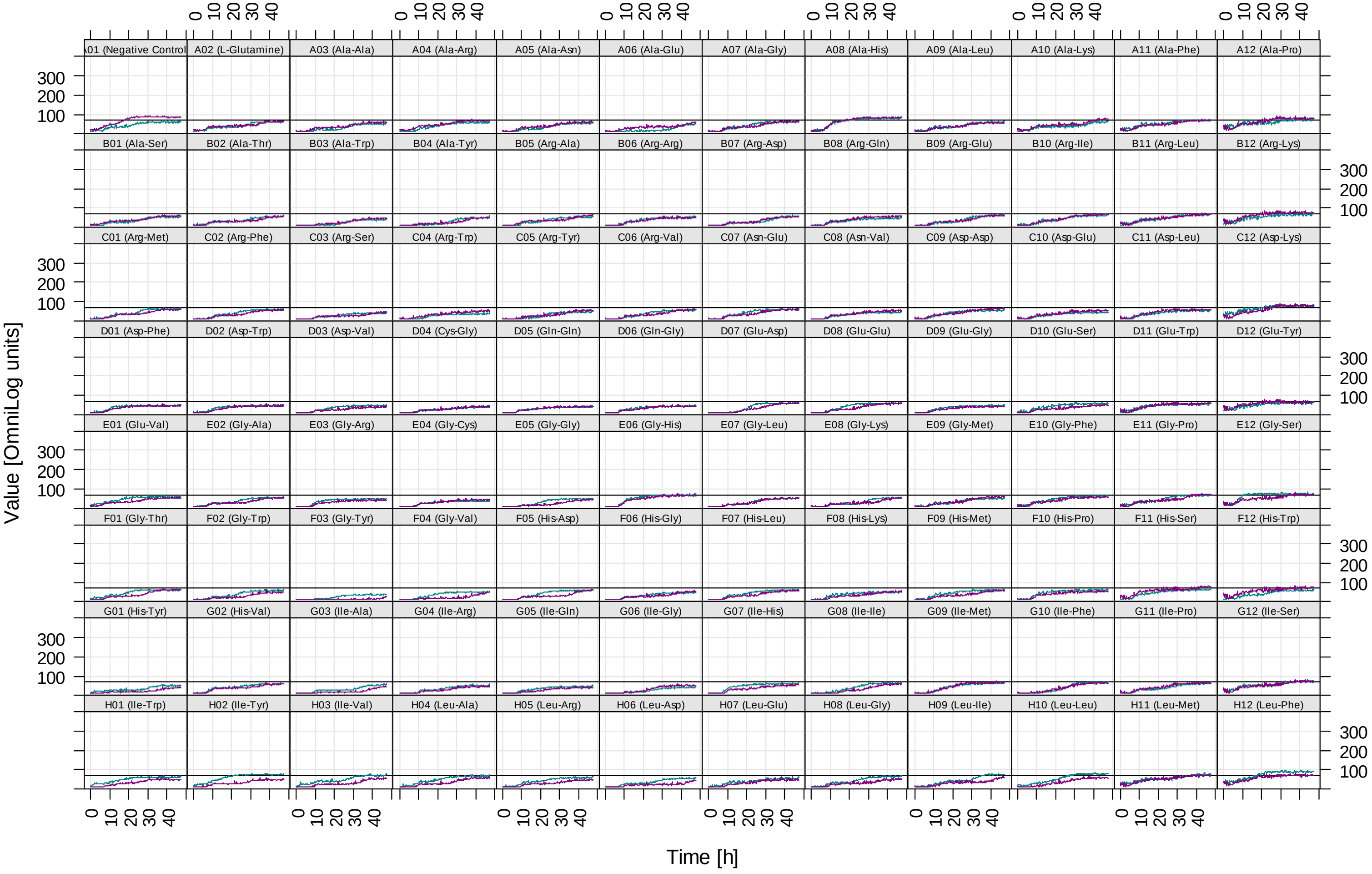

## PM07 (Peptide Nitrogen Sources)

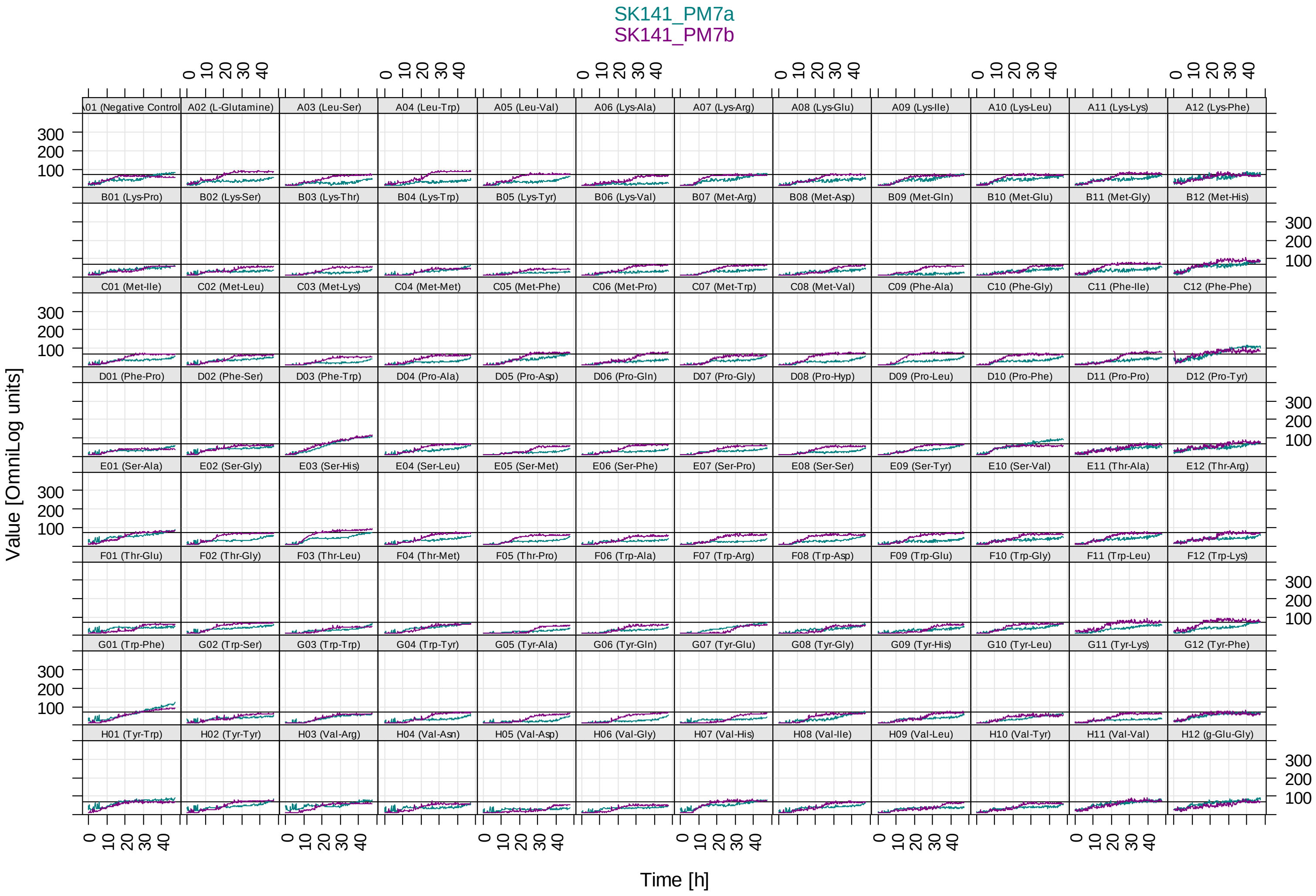

# PM08 (Peptide Nitrogen Sources)

SK141_PM8a
SK141_PM8b

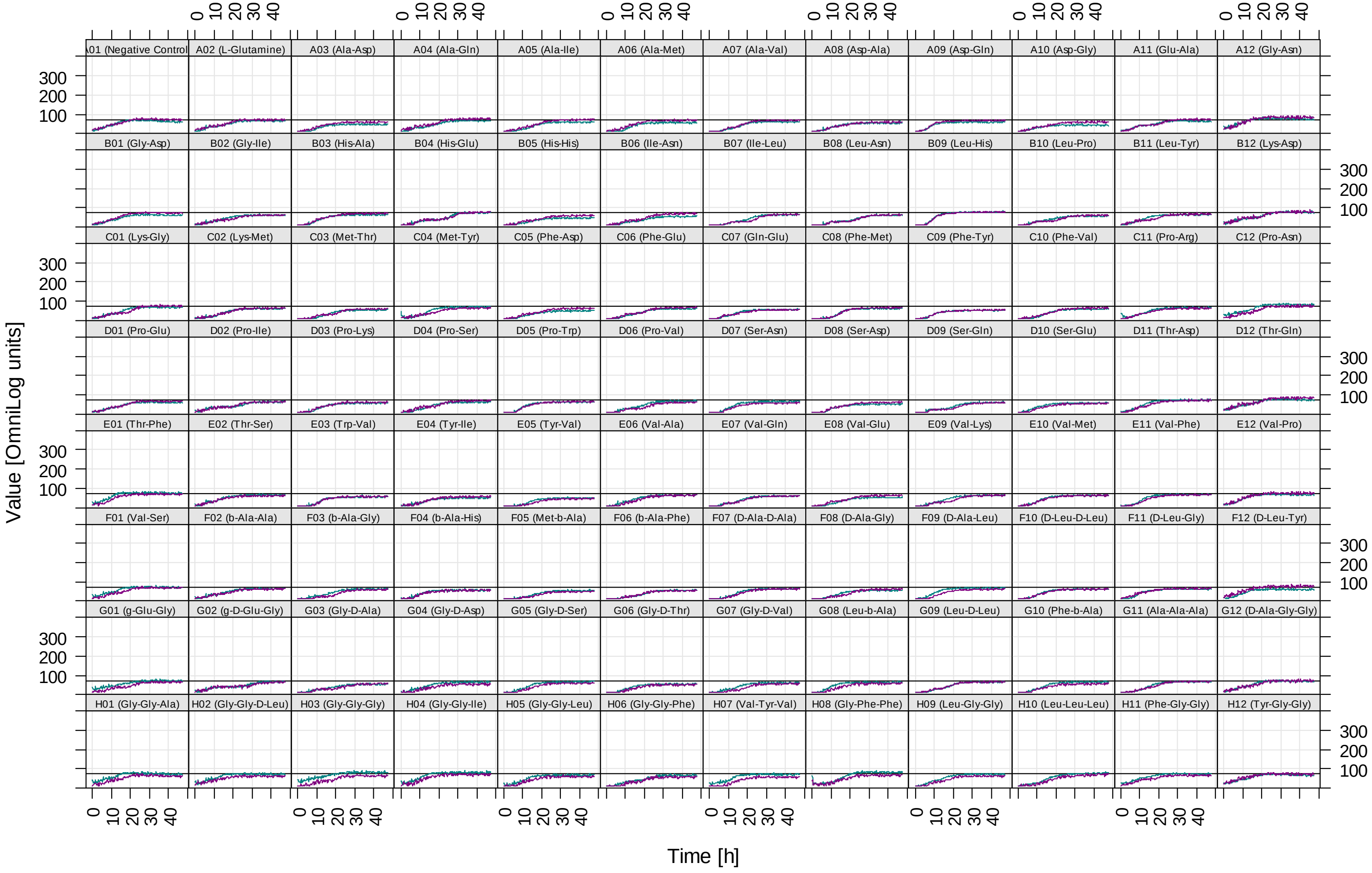

## PM09 (Osmolytes)

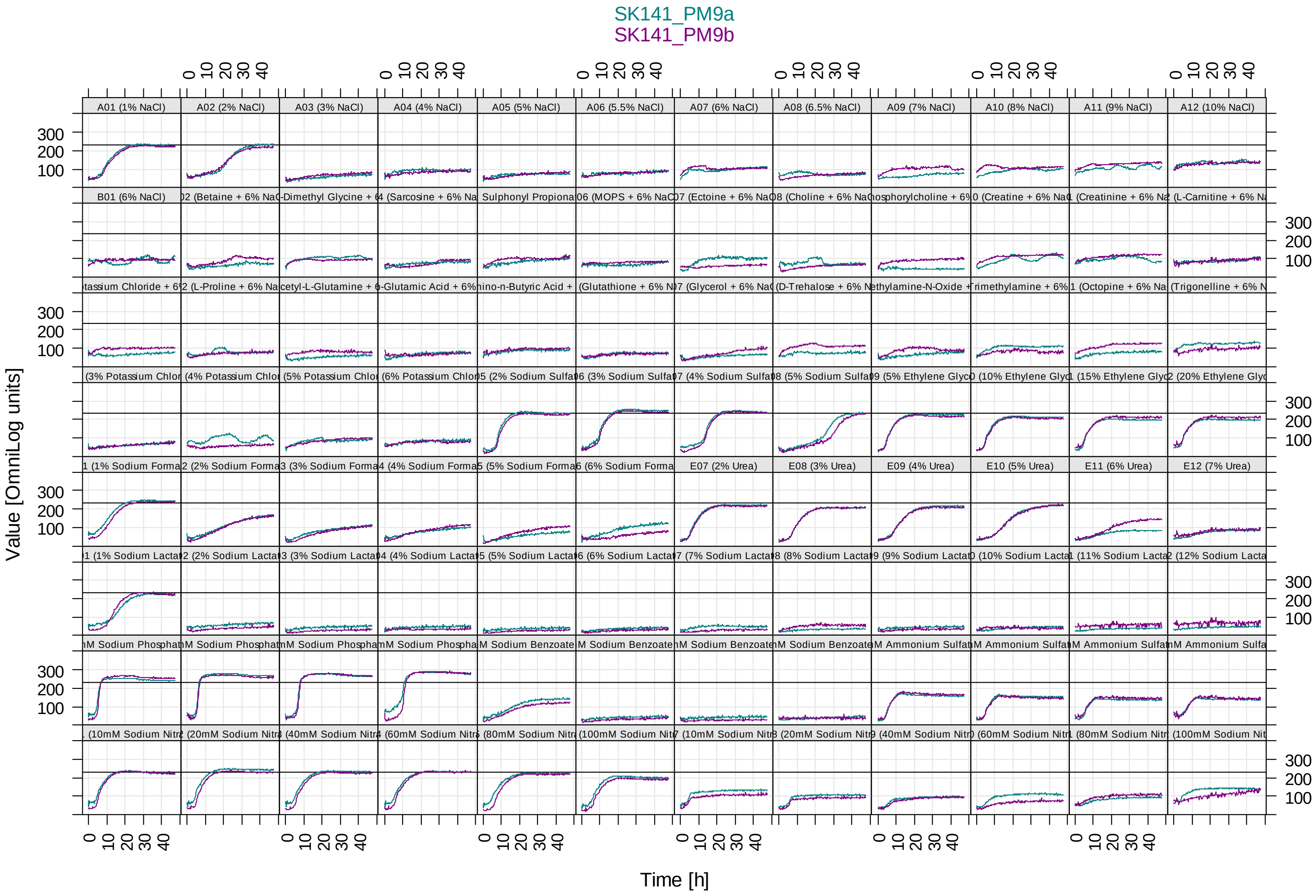

# PM10 (pH)

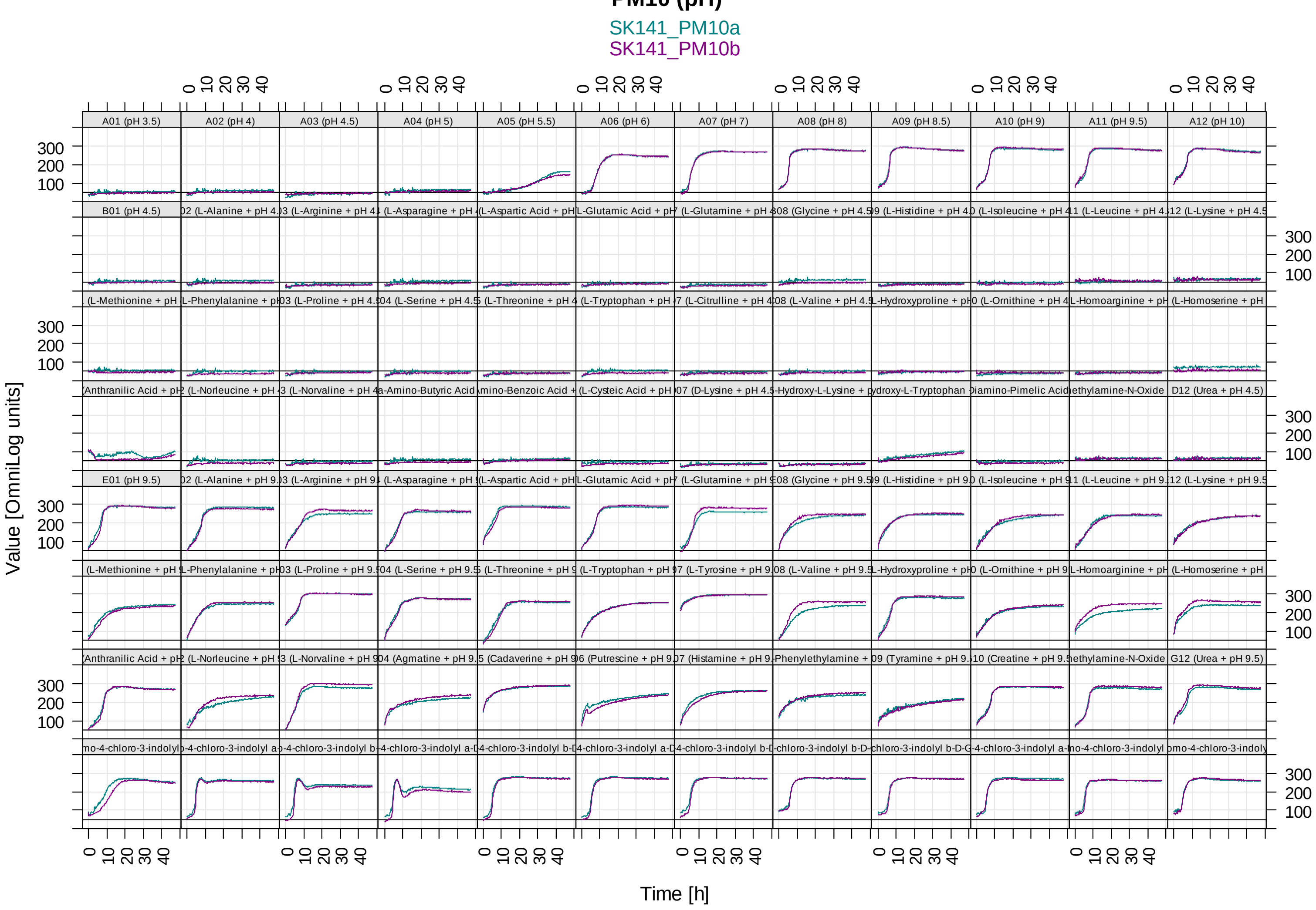

Supplement: Data Sheet S3 — Biolog results for S. oralis SK141. [file DataSheet_3.pdf]
